# Privacy Policy Compliance of Chronic Disease Management Apps in China: Scale Development and Content Evaluation

**DOI:** 10.2196/23409

**Published:** 2021-01-28

**Authors:** Zhenni Ni, Yiying Wang, Yuxing Qian

**Affiliations:** 1 School of Information Management Wuhan University Wuhan China; 2 Center for Studies of Information Resources Wuhan University Wuhan China; 3 Big Data Institute Wuhan University Wuhan China; 4 School of International Law Shanghai University of Political Science and Law Shanghai China

**Keywords:** mHealth, noncommunicable diseases, content analysis

## Abstract

**Background:**

With the development of mobile health (mHealth), chronic disease management apps have brought not only the possibility of reducing the burden of chronic diseases but also huge privacy risks to patients’ health data.

**Objective:**

The purpose of the study was to analyze the extent to which chronic disease management apps in China comply with the Personal Information Security Specification (PI Specification).

**Methods:**

The compliance of 45 popular chronic disease management apps was evaluated from the perspective of the information life cycle. To conduct a fine-grained evaluation, a scale based on the PI Specification was developed. Finally, 6 level 1 indicators, 22 level 2 indicators, and 61 level 3 indicators were defined.

**Results:**

There were 33/45 apps (73%) with a privacy policy, and the average score of these apps was 40.4 out of 100. Items of level 1 indicators with high scores included general characteristics (mean 51.9% [SD 28.1%]), information collection and use (mean 51.1% [SD 36.7%]), and information sharing and transfer (mean 50.3% [SD 33.5%]). Information storage and protection had the lowest compliance with PI Specification (mean 29.4% [SD 32.4%]). Few personal information (PI) controllers have stated how to handle security incidents, including security incident reporting (7/33, 21%), security incident notification (10/33, 30%), and commitment to bear corresponding legal responsibility for PI security incidents (1/33, 3%). The performance of apps in the stage of information destruction (mean 31.8% [SD 40.0%]) was poor, and only 21% (7/33) apps would notify third parties to promptly delete PI after individuals cancelled their accounts. Moreover, the scoring rate for rights of PI subjects is generally low (mean 31.2% [SD 35.5%]), especially for obtaining copies of PI (15%) and responding to requests (25%).

**Conclusions:**

Although most chronic disease management apps had a privacy policy, the total compliance rate of the policy content was low, especially in the stage of information storage and protection. Thus, the field has a long way to go with regard to compliance around personal privacy protection in China.

## Introduction

### Background

Chronic diseases, such as diabetes and hypertension, are a major global health issue affecting many countries [1]. Fortunately, the booming of mobile health (mHealth) offers opportunities for chronic diseases prevention, treatment, and daily self-management. The health benefits of mHealth interventions for patients with chronic diseases have been demonstrated [2]; mHealth apps can be used to collect and monitor health data [3,4], promote and support self-management [5,6], and provide medication and appointment reminders [7]. Different from other types of mHealth apps, such as online registration and online consultation, chronic disease management apps allow individuals to generate large quantities of data about their lifestyle, introducing risks to the security and privacy of patient data.

Considering the potential negative effects of security breaches of health data systems, such as social stigma, damage to reputation, and fraud in the health system [8], privacy has become an important factor discouraging patients from using mHealth apps for health care [9-11]. Different from other kinds of disease management, the care of chronic diseases requires patients to regularly track key health indicators. It means that protecting the safety and privacy of personal information (PI) is crucial for chronic disease management apps. A few studies were related to the privacy policy of chronic disease management apps. These studies mainly involved 3 focal aspects: the quality assessment [12,13], the complexity analysis of app privacy policies [14], and the security analysis [15]. Although the above 3 aspects involved privacy policies, the evaluations were relatively rough.

As for the evaluation criteria, various standards were used to evaluate the privacy of mHealth apps. Most papers established evaluation indicators based on the existing literature [16,17] or authors’ criteria [18-20]. The most common items in the evaluation criteria included stating processing purposes, determining the recipient of personal data, the existence of the data rights of the individuals, and the existence of privacy policies. Although a few papers on the privacy assessment of mHealth apps were based on laws or regulations, such as General Data Protection Regulation (GDPR), Fair Information Practices (FIPS) [18,21], some of them proposed a set of items to check the compliance of laws or regulations [20,22].

In China, the Information Security Technology–Personal Information Security Specification (GB/*t* 35273-2020) (PI Specification) came into effect on October 1, 2020 [23]. This specification, also as the standard basis for apps’ privacy certification, lays out granular guidelines for how personal data should be collected, used, and shared. Besides, it provides a template of PI protection policy in the form of attachments. Although PI Specification is a national voluntary standard instead of a mandatory standard, it provides a reference for the industry. However, the compliance with PI Specification of mHealth apps remains unclear. In each step of the information life cycle, the patient’s PI is at risk of leakage, such as collection, storage, usage, sharing, destruction, and so on. Therefore, it is necessary to review the compliance of the privacy policy of mHealth apps based on PI Specification from various stages of the information life cycle, especially for chronic disease management apps that have insufficient privacy assessment.

### Objectives

This study aimed to evaluate the compliance of privacy policies of chronic disease apps with the PI Specification from the perspective of the information life cycle. Specifically, this study can provide answers to the following 2 research questions: (1) To what extent do chronic disease apps comply with PI Specification 2020? (2) Among the various stages of the information life cycle, which stage has the weakest privacy policy protection?

## Methods

### Apps Selection

Considering the popularity of Android in China [24], this study investigated mHealth apps in Android app stores. The top 4 Android app stores were selected, which accounts for 61.0% of the Chinese Android market [25], including Tencent My App (26.0%) [26], Huawei App Market (15.1%) [27], Oppo Software Store (10.2%) [28], and 360 Mobile Assistant (9.7%) [29]. The apps returned by queries for “noncommunicable diseases,” “chronic disease,” “diabetes,” “blood pressure,” “hypertension,” “heart disease,” “kidney,” “cardiovascular,” “asthma,” “respiratory disease,” or “cancer” were included in the set of chronic diseases management apps.

This search was conducted on October 2, 2020. Our sample was filtered based on the title and description in the app stores. The app met inclusion criteria if it (1) was in Chinese; (2) required the input of PI over time; (3) had the general public as its target user group rather than clinicians; and (4) had over 100,000 downloads. The authors saved all privacy policies as text files and recorded the downloads, update time, and disease category.

**Figure 1:**
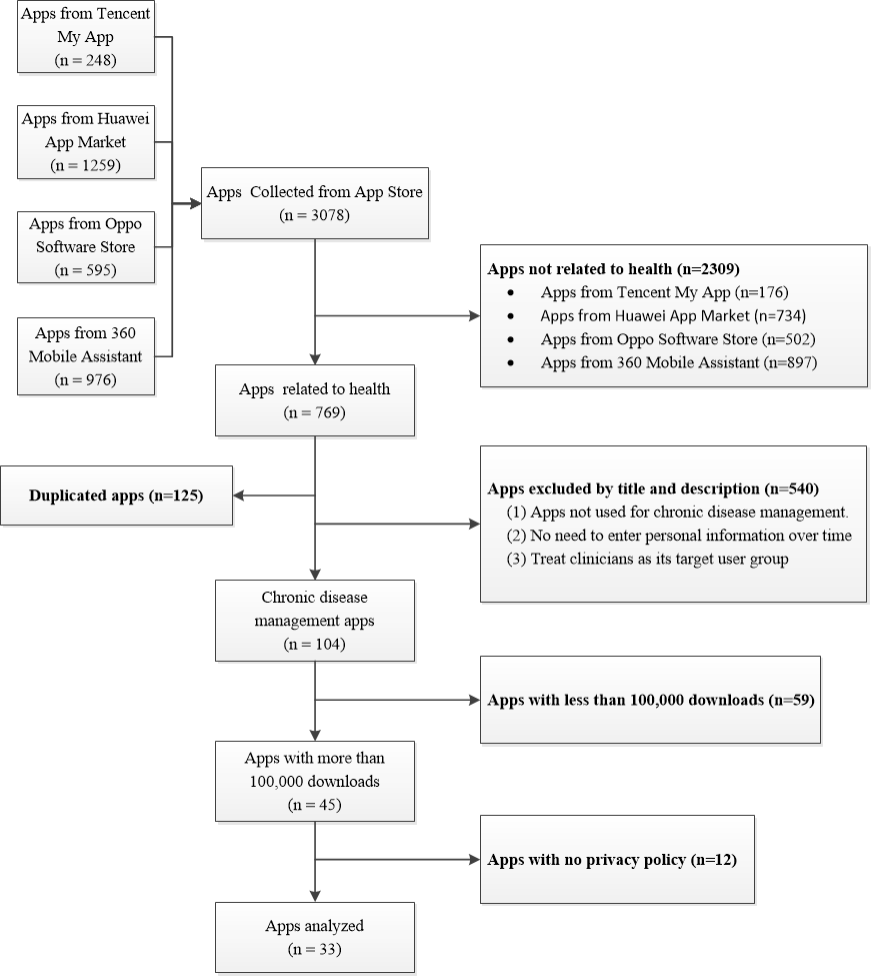
Flow chart of the search strategy.

A total of 45 apps met the inclusion criteria (Figure 1). Among them, 12/45 apps (27%) had no privacy policy. Excluding apps without a privacy policy, the remaining 33 privacy policies were analyzed.

### Scale Development and Scoring

The level 1 and level 2 evaluation indicators are shown in Textbox 1. Level 3 evaluation indicators for privacy policies are listed in Multimedia Appendix 1. Based on the information life cycle, 6 level 1 indicators were developed, including information collection and use, information storage and protection, information sharing and transfer, information destruction, general characteristics of privacy policies, and rights of PI subjects. There are 22 items on level 2 indicators and 61 items on level 3 indicators. For each level 3 indicator, a brief explanation, example sentences, and corresponding clauses of PI Specification are listed in Multimedia Appendix 2.

Each level 3 indicator was scored as 1 point if the privacy policy complies with the indicator and 0 otherwise. Scoring rate of each level 3 indicator was defined as the percentage of the number of apps scored 1 point in the total sample. Scoring rate of each level 2 indicator was the average of all level 3 indicators under that level 2 indicator. Scoring rate of each level 1 indicator, which indicated the compliance of apps in the corresponding stage of the information life cycle, was the average of all level 2 indicators under that level 1 indicator. For each app, the sum of all level 3 indicators scores was converted into a percentage system as a final score; the final score represented the compliance of the app. Bar graphs are used to visualize the degree of policy compliance. The ordinate of bar graphs is the scoring indicators, including level 3 and level 2 indicators, and the scoring rate of level 2 indicators. The abscissa is the scoring rate of level 3 indicators. In order to more intuitively reflect the scores of the level 2 indicators, we use different colors to visualize each level 2 indicator; if the scoring rate is close to the average score, it is yellow; if the scoring rate is close to the minimum value, it is red; If the scoring rate is close to the maximum value, it is green.

Initially, 2 raters (ZN and YW) independently reviewed 21% (7/33) of randomly selected apps to assess the level of agreement; the Kappa-Cohen Index was 0.87, which denoted an almost perfect agreement. Then, 2 raters (ZN and YW) discussed indicators with inconsistent scores, and each rater analyzed half of the remaining apps after the standard was unified.

**Level 1 and level 2 evaluation indicators for privacy policies.**

**1. General characteristics**

- App scope
- Policy disclosure
- Policy updates

**2. Information collection and use**

- Information collection and usage rules for business functions
- Personal sensitive information

**3. Information storage and protection**

- Storage security
- The handling of security incidents

**4. Information sharing and transfer**

- Entrusted processing
- Sharing of PI
- Transfer of PI
- Public disclosure of PI
- Cross-border transmission

**5. Information destruction**

- Storage time limit
- Data deletion and anonymization

**6. Rights of PI subjects**

## Results

### Sample Distribution

The basic characteristics of these apps are presented in Table 1. The types of chronic diseases targeted by apps mainly include diabetes (11/45, 24%), hypertension (4/45, 9%), heart disease (4/45, 9%), cancer (2/45, 4%), and comprehensive chronic disease management (19/45,42%). The comprehensive chronic disease management app referred to providing users with long-term, multifaceted chronic disease prevention and treatment services that were not targeted at specific chronic disease. Besides, it included a small number of apps for other types of chronic diseases (5/45, 11%), such as asthma, chronic kidney disease, and chronic skeletal muscle diseases. Most apps (30/45, 67%) had between 100,000 and 1,000,000 downloads; 73% (33/45) of apps were updated in 2020.

**Table 1:** Sample distribution of chronic disease management apps (N=45).

Category		Count, n (%)
**Disease category**		
	Diabetes	11 (24)
	Hypertension	4 (9)
	Heart disease	4 (9)
	Cancer	2 (4)
	Comprehensive	19 (42)
	Others	5 (11)
**Downloads**		
	100,000-1,000,000	30 (67)
	1,000,000-10,000,000	11 (24)
	10,000,000	4 (9)
**Updated**		
	2014-2016	2 (4)
	2017-2019	10 (22)
	2020	33 (73)

### Compliance Evaluation

**Figure 2:**
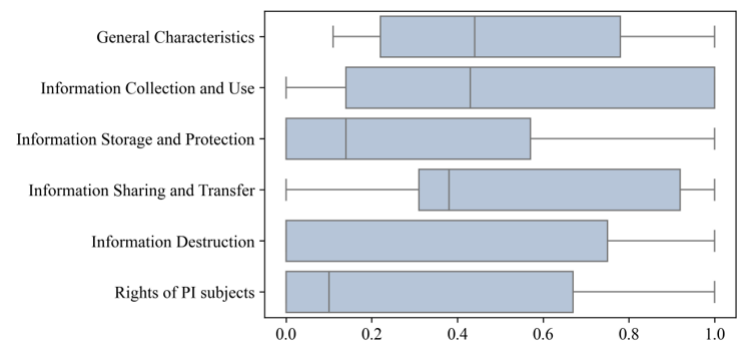
The scoring rate of chronic disease management apps on level 1 indicators. PI: personal information.

The average score of 33 apps was 40.4 out of 100, and the degree of dispersion was very high (SD 31.2). The evaluation results on level 1 indicators of privacy policies are presented in Figure 2. The most complied-with items in level 1 indicators were the following: general characteristics (mean 51.9% [SD 28.1%]), information collection and use (mean 51.1% [SD 36.7%]), and information sharing and transfer (mean 50.3% [SD 33.5%]). However, some indicators had a low degree of overall compliance, such as information storage and protection (mean 29.4% [SD 32.4%]), information destruction (mean 31.8% [SD 40.0%]), and rights of PI subjects (mean 31.2% [SD 35.5%]). The name and evaluation results of each app are listed in Multimedia Appendix 3.

The scoring rate for level 2 indicators ranged from 15.2% to 75.8%, with an average of 40.4%. We visualized the evaluation results with bar graphs, in which the color of bars indicates the scoring rate of level 2 indicators (the value in parentheses) and the length of bars indicates the scoring rate of level 3 indicators.

**Figure 3:**
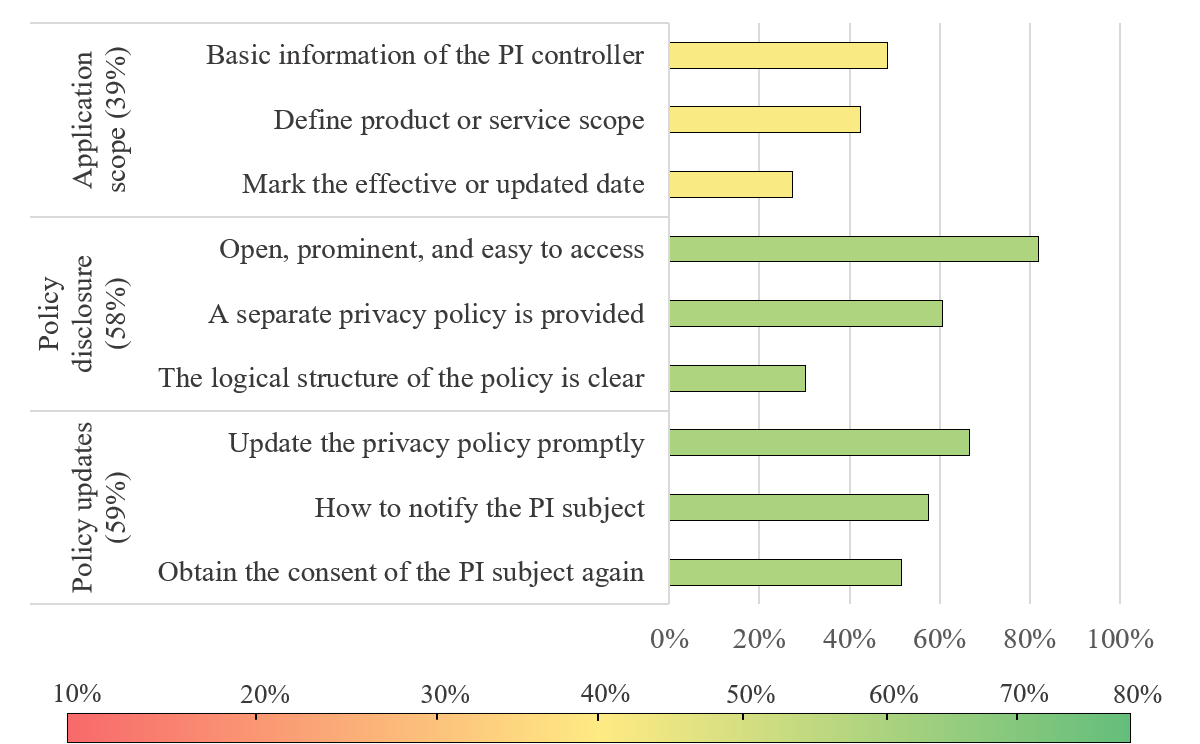
Compliance evaluation results of the privacy policies general characteristics. PI: personal information.

The general characteristics of privacy policy reflect its openness, readability, and timeliness of updates. Compliance evaluation results of the privacy policies general characteristics are shown in Figure 3. Some level 2 indicators scored high, such as policy updates (59%) and disclosure (58%). More than one-half of the apps promised to notify users (19/33, 58%) and obtain the explicit consent of PI subjects again (17/33, 52%) if the policy was updated. As for policy disclosure, although most apps provided independent (20/33, 61%) and easily accessible (27/33, 82%) privacy policies, only a few apps (10/33, 30%) had a clear logical structure and provided a directory summary. In terms of scope, a few apps (9/33, 27%) marked the update date or effective time of the privacy policy, which indicated that the timeliness of policy updates was low.

**Figure 4:**
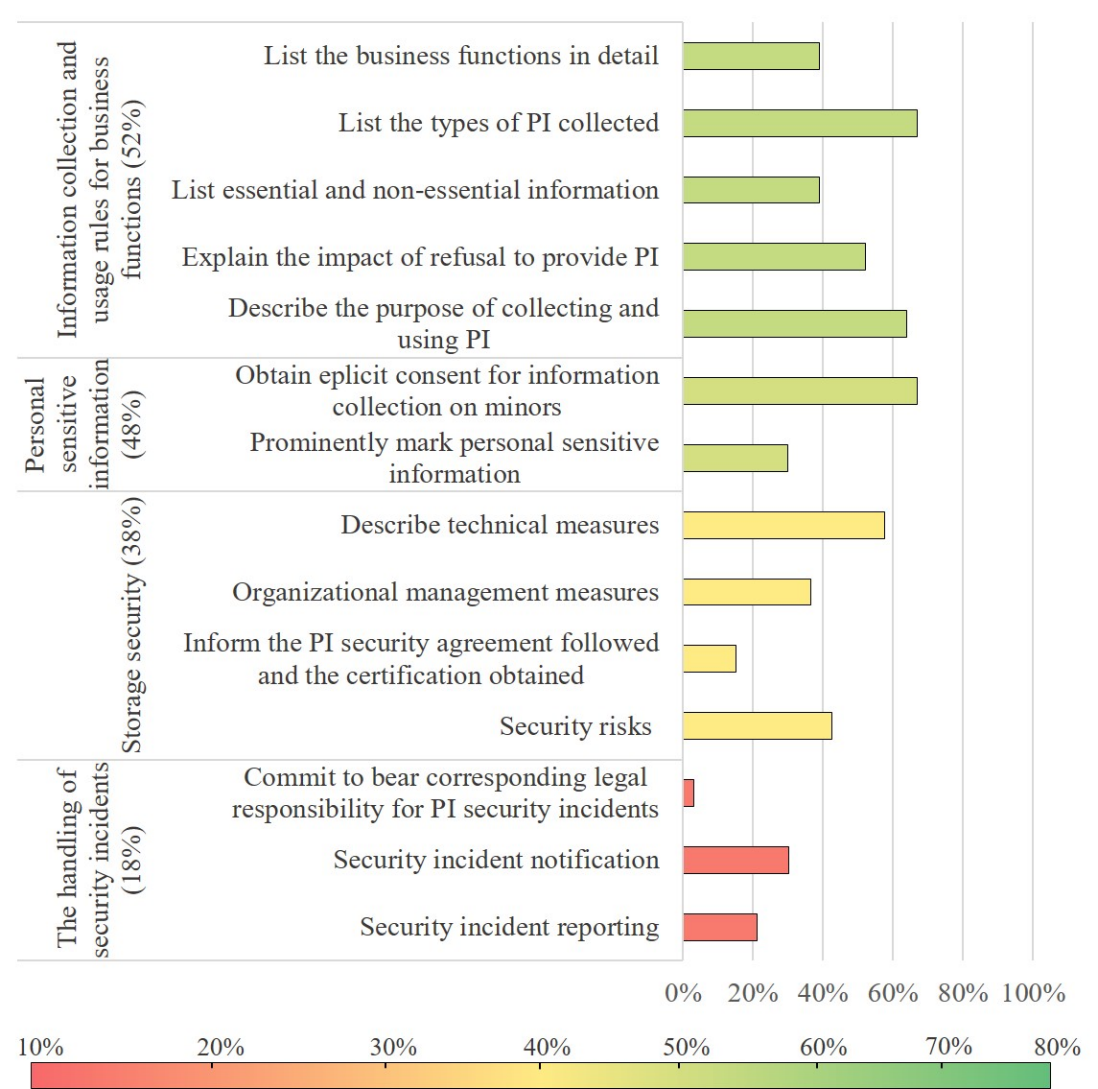
Evaluation results in the stage of information collection and use, and the stage of information storage and protection. PI: personal information.

Compliance evaluation results in the stage of information collection and use, and the stage of information storage and protection are presented in Figure 4. In the information collection and use stage, the scoring rate of all level 2 indicators reached the average, and the overall compliance degree was relatively high. Because the research object of this article was chronic disease management apps, all apps in this research involved the collection and processing of personally sensitive information. However, in terms of personal sensitive information, only 30% (10/33) of apps marked personal sensitive information prominently.

Although the compliance level of storage security was close to the average (38%), most apps (28/33, 85%) did not inform PI subjects the security agreement they followed and the certification they obtained. The compliance level of the handling of security incidents (18%) was far below the average. Among the 33 apps, only 1 app (3%) promised to bear corresponding responsibilities if a security incident occurred. In addition, no more than one-third of apps described how to inform PI subjects after a security incident (10/33, 30%), and whether they would report it truthfully to government organizations (7/33, 21%).

**Figure 5:**
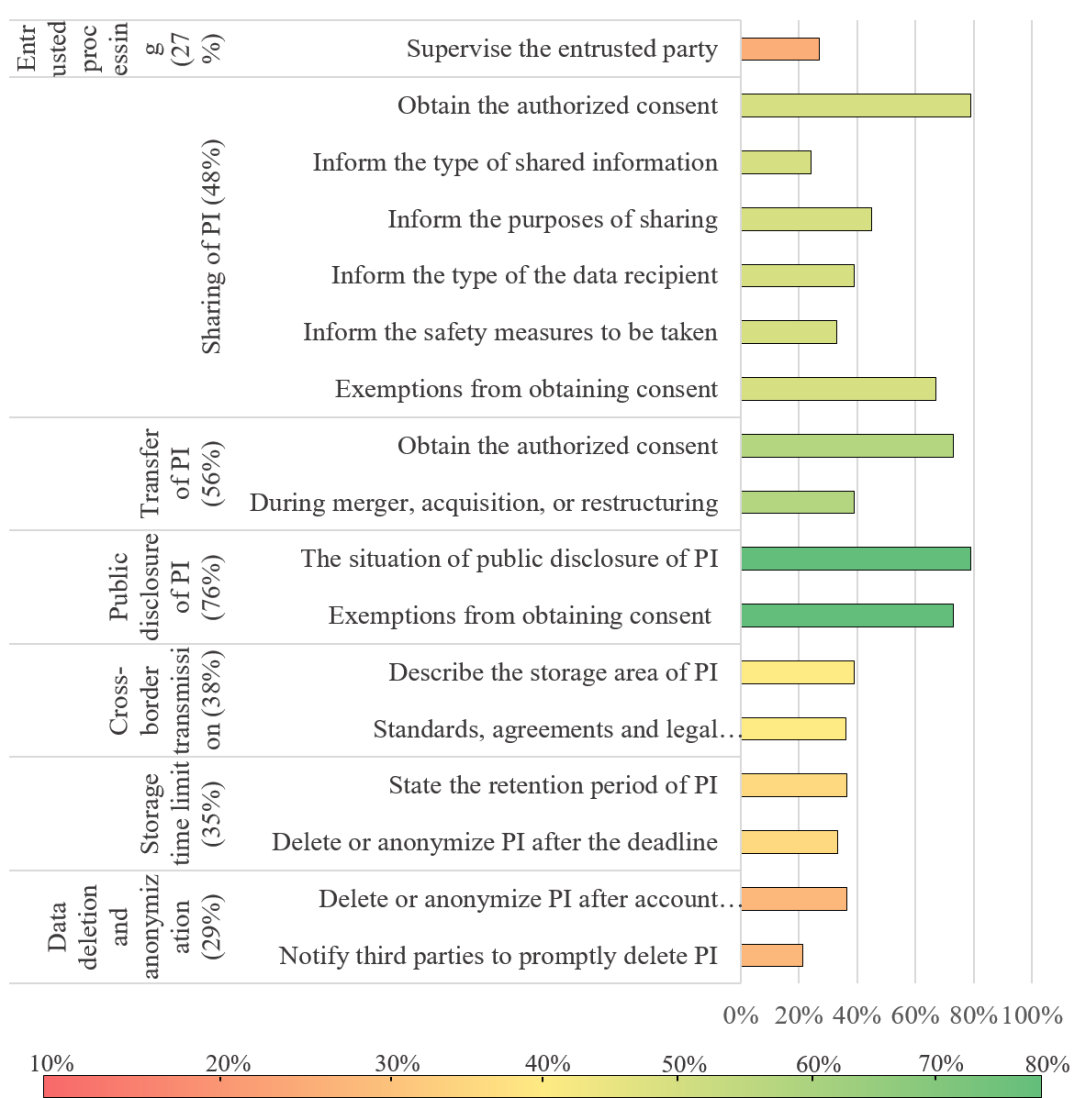
Evaluation results in the stage of information sharing and transfer, and the stage of information destruction. PI: personal information.

Compliance evaluation results in the stage of information sharing and transfer, and the stage of information destruction are shown in Figure 5. Only 24% (8/33) of apps informed the type of shared information and 33% (11/33) of apps informed the security measures taken before sharing, such as anonymization or deidentification. Entrusted processing scored low; only 27% (9/33) of apps stated that they would supervise the entrusted party by establishing the third-party’s responsibilities and duties through contract or other such means. The 2 level 2 indicators of the information destruction stage, namely, storage time limit (35%) and data deletion and anonymization (29%), were all lower than the average scoring rate. Especially if PI subjects request to delete user data, only 21% (7/33) of apps would notify third parties to promptly delete their PI.

**Figure 6:**
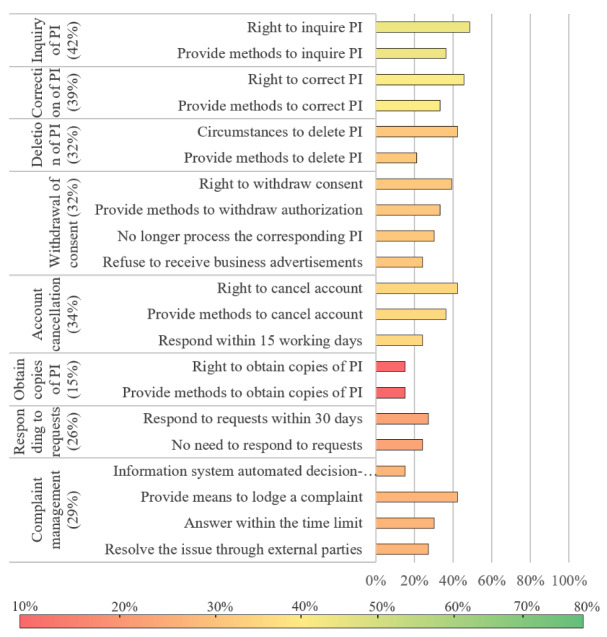
Compliance evaluation results of the right of PI subjects. PI: personal information.

Most privacy policies had a low scoring rate for the indicators related to rights of PI subjects (Figure 6), especially the right to obtain a copy of PI, which was only 15% (5/33). Scores for level 2 indicators such as complaint management (29%) and responding to requests (26%) were far below the average, which meant that most apps did not pay attention to the handling of user requests and complaints.

## Discussion

### Key Findings

In this study, we proposed a scale based on PI Specification 2020 for assessing the compliance of China’s chronic disease apps privacy policies from various stages of the information life cycle. Fu and Zhao [30] analyzed the privacy policies of 20 mHealth apps in China based on PI Specification 2017. In their study, the privacy policies were analyzed from 6 aspects, including information collection, cookies and other related technologies, PI storage and protection, PI sharing, PI processing rights, and minor information protection. However, their study did not conduct a fine-grained quantitative analysis and evaluation of each item and it could not reveal the app’s compliance with specific articles in PI Specification. In this paper, 6 level 1 indicators, 22 level 2 indicators, and 61 level 3 indicators were defined and a fine-grained evaluation was conducted. PI controllers and subjects can use the scale to obtain a percentual score that defines the compliance of privacy policies.

According to the results, most of the apps collected in the initial sample (33/45, 73%) included a privacy policy, which was similar to a previous assessment of cancer apps by Benjumea et al [22] who found that 71% of the apps in their sample had a privacy policy. Considering that the prevalence of privacy policies for high-download apps is significantly higher than that of low-download apps (high downloads: 15/17, 88%; and low downloads: 33/64, 52%; *P*=.006) [31], our result might be higher than the actual situation.

Regarding scores, only 39% (13/33) of apps in our sample had a score greater than or equal to 40 points, with an average score of 40.4 out of 100 (SD 31.2), which indicated that the majority of chronic disease management apps in China had low compliance with PI Specification 2020. This result is consistent with the prior finding by Fu and Zhao [30], who determined that most mHealth apps in China did not meet the requirements of PI Specification. Benjumea et al [22] analyzed the privacy policies of 31 cancer Android apps from the Google Play website (Spanish version) and obtained an average score of 50.5 points; in Hutton et al [21], the average score for 64 self-tracking mHealth apps from Google Play was 46.2% (SD 24.3%). These differences might also be the result of different mHealth app types, evaluation scales, and even normative background (Hutton et al [21] refer to GDPR, FIPS, and usability, whereas Benjumea et al [22] refer to GDPR). What we compared is the degree to which apps complied with local laws or regulations, rather than the degree to which they protected the privacy of users. Thus, according to the evaluation results, the compliance of chronic disease management apps in China Android app stores might be slightly lower than that of mHealth apps in Google Play.

In terms of general characteristics, policy disclosure and policy updates are the basic prerequisites for a privacy policy to effectively protect the legal rights of PI subjects. The level 3 indicators under policy disclosure and policy updates can maintain a scoring rate of 57%-58%, which indicated that most PI controllers had a basic awareness of protecting user privacy. However, only 48% (16/33) of apps introduce the basic information of the PI controller in the privacy policies, which is far lower than a previous study (77%) [22].

In the stage of information collection and use, 64% (21/33) of apps stated the purpose of collecting and using PI, which was in line with the result (61%) of Hutton et al [21]; 52% (17/33) of apps described the impact of refusal to provide PI, which was far higher than the result (27%) of Benjumea et al [22]. According to PI Specification Article 5.5, if the app involves the collection of personal sensitive information, the PI controller should clearly mark or highlight the information. However, only 30% (10/33) of apps prominently marked personal sensitive information in their privacy policies.

Information sharing has always been a hotspot in privacy policy analysis. Robillard et al [32] found that 68% of privacy policies stated that users’ PI may be shared with third parties, whereas only 10% of apps stated that users’ PI would not be shared without their consent. In this paper, the majority of apps with a privacy policy that we assessed were highly compliant with PI Specification in data sharing (48%), transmission (56%), and public disclosure (76%). In terms of the consent of PI subjects, considerable proportions of privacy policies mentioned that they would obtain the consent of PI subjects before sharing (26/33, 79%), transfer (24/33, 73%), and public disclosure (24/33, 73%) PI. While most apps would obtain the consent of PI subjects before sharing PI, no more than one-fourth of apps informed the type of PI they would share. Furthermore, during the information sharing and transfer stage, the most worrying issue was the lack of safety measures (11/33, 33%) and supervision of third parties (9/33, 27%), which brought serious security risks to PI of patients.

Among the stages of the information life cycle, the stage of information storage and protection had the lowest compliance with PI Specification. According to Zhou et al [11], most users did have concerns about their privacy when using mHealth apps and expected the apps to take a variety of security measures, such as regular password updates, remote wipe, user consent, and access control. However, according to our assessment, approximately two-thirds of chronic disease management apps lacked the description of security measures in the level of organization management. Concerningly, only few PI controllers (18%) have stated how to handle security incidents, such as security incident reporting, security incident notification, and commitment to bear corresponding legal responsibility for PI security incidents.

The timely destruction of PI is essential to the privacy of patients. Few privacy policies complied with PI Specification in terms of the storage time limit (35%) and the deletion or anonymization of PI after account cancellation (29%). One noteworthy point here was that only 21% (7/33) of chronic disease management apps would notify third parties to promptly delete PI after PI subjects cancelled their accounts. According to PI Specification [23], the PI retention period should be the shortest time needed to achieve the purpose (Article 6.1); after the retention period is exceeded or the account is cancelled, PI controllers should carry out data deletion or anonymization (Article 6.1, Article 8.5). Judging from the assessment results of this study, the performance of apps in the stage of information destruction was far from reaching the requirements of PI Specification.

The scoring rate for rights of PI subjects is generally low, especially for obtaining copies of PI (15%) and responding to requests (25%), which was consistent with a previous study [21]. Furthermore, during our evaluation, we noticed that compared with the description of rights of PI subjects, the scoring rate of how to exercise rights of PI subjects is usually lower. For example, 48% (16/33) of apps stated the right of PI inquiry, whereas only 36% (12/33) of apps provided methods to inquire PI. These findings demonstrated that most Android chronic disease management apps in China can hardly guarantee the exercise of patients’ rights.

### Implications and Recommendations

The contributions of this study are threefold. First, we developed a new scale based on PI Specification. From the perspective of information life cycle management, the compliance of privacy policies can be evaluated systematically, and the scale can be generalizable to other kinds of apps in China. Based on our scale, app operators can also conduct a fine-grained self-assessment of their app privacy policies. Second, through the analysis of privacy policies, physicians and patients could better understand what information patients provide to the app companies and the potential risk of providing this information to non–health care providers, especially in terms of information storage and protection. Moreover, we investigated and assessed the current state of practice in chronic disease management apps regarding the protection of health-related data. The indicators in this paper were based on the PI Specification 2020, and findings presented in this article could provide insights into the implementation of the new specification in China. Personal health information is highly sensitive and the leakage of daily health data may cause negative effects [8]. In this regard, we would like to make the following recommendations:

First, improve the readability of the privacy policy. The results from a 2018 study [33] suggested that privacy policies are not comprehensible to most adults. Thus, it is of great significance for apps to make their privacy policies shorter and simpler so that PI subjects can understand it. Second, strengthen government supervision and industry self-regulation. The Personal Information Protection Law of the People’s Republic of China (Draft Law) was released for seeking opinions from the public on October 21, 2020 [34]. Different from the PI Specification, which is a national recommended standard instead of a mandatory standard, the promulgation and implementation of the Personal Information Protection Law will provide strong legal support for the protection of personal privacy and user rights. Moreover, it is important to pay attention to the positive effects of mHealth industry self-discipline and encourage mHealth industry organizations to draft industry rules to collect and use personal health information legally.

### Limitations

First, our indicators may not be practical for apps in some special cases. For example, all level 2 indicators under “Sharing of PI” cannot be evaluated if the app does not share any PI. Assigning 1 point or 0 points, in this case, would overrate or underrate the privacy policy, respectively. Second, although we assessed the compliance of the privacy policies, we did not conduct a technical audit to evaluate if the data handling procedures outlined in the policy are implemented. It is reported that the disclosures regarding third-party data transmission do not match actual behavior [16]. Thus, future work can explore the correspondence between privacy disclosures and how apps for chronic disease handle personal data.

### Conclusions

Despite these limitations, our findings demonstrated a general lack of compliance regarding the handling of users’ health data submitted to chronic disease management apps. Although most chronic disease management apps had a privacy policy, the total compliance rate of the policy content was low. In addition, few apps could handle security incidents according to the requirements of PI specification. Importantly, it was difficult for PI subjects to exercise their rights in accordance with the privacy policies, especially in the stage of information destruction. Overall, our findings suggest the field has a long way to go with regard to compliance around data handling in China. Only by calling attention to this large need, can we change the practices and create a safer online environment for users’ daily health information.

## Appendix

Multimedia Appendix 1 Evaluation indicators for privacy policies.

Multimedia Appendix 2 Evaluation guide.

Multimedia Appendix 3 List of mHealth apps names and evaluation results.

## Abbreviations

FIPS: Fair Information Practices
GDPR: General Data Protection Regulation
mHealth: mobile Health
PI: personal information
PI Specification: Information Security Technology–Personal Information Security Specification (GB/*t* 35273-2020)
