# Supplementary material for: Privacy Policy Compliance of Chronic Disease Management Apps in China: Scale Development and Content Evaluation
Source: JMIR Mhealth Uhealth. 2021 Jan 28;9(1):e23409. doi: 10.2196/23409 (PMC7878107; doi:10.2196/23409)
Supplement: Multimedia Appendix 1 [file mhealth_v9i1e23409_app1.docx]

| Level 1 indicator | Level 2 indicator | Level 3 indicator |
| --- | --- | --- |
| **General characteristics** |  |  |
|  | **App scope** |  |
|  |  | Introduce the basic information of the PI controller |
|  |  | Define product or service scope |
|  |  | Mark the effective or updated date |
|  | **Policy disclosure** |  |
|  |  | The policy is open, prominent, and easy to access |
|  |  | A separate privacy policy is provided |
|  |  | The logical structure of the policy is clear |
|  | **Policy updates** |  |
|  |  | Update the privacy policy promptly |
|  |  | Explain the method used to notify the PI subject |
|  |  | Obtain the consent of the PI subject again |
| **Information collection and use** |  |  |
|  | **Information collection and usage rules for business functions** |  |
|  |  | List the business functions in detail |
|  |  | List the types of PI collected |
|  |  | List essential and non-essential information |
|  |  | Explain the impact of refusal to provide PI |
|  |  | Describe the purpose of collecting and using PI |
|  | **Personal sensitive information** |  |
|  |  | Obtain explicit consent for information collection on minors |
|  |  | Prominently mark personal sensitive information |
| **Information storage and protection** |  |  |
|  | **Storage security** |  |
|  |  | Describe technical measures |
|  |  | Describe organizational management measures |
|  |  | Inform the PI security agreement followed and the certification obtained |
|  |  | Describe the security risks |
|  | **The handling of security incidents** |  |
|  |  | Commit to bear corresponding legal responsibility for PI security incidents |
|  |  | Security incident notification |
|  |  | Security incident reporting |
| **Information sharing and transfer** |  |  |
|  | **Entrusted processing** |  |
|  |  | Supervise the entrusted party |
|  | **Sharing of PI** |  |
|  |  | Obtain the authorized consent when sharing PI |
|  |  | Inform the type of shared information |
|  |  | Inform the purposes of sharing |
|  |  | Inform the type of the data recipient |
|  |  | Inform the safety measures to be taken prior to sharing PI |
|  |  | Exemptions from obtaining authorized consent prior to sharing PI |
|  | **Transfer of PI** |  |
|  |  | Obtain the authorized consent when transferring PI |
|  |  | Transfer rules of PI during merger, acquisition, or restructuring |
|  | **Public disclosure of PI** |  |
|  |  | The situation of public disclosure of PI |
|  |  | Exemptions from obtaining authorized consent prior to public disclosure PI |
|  | **Cross-border transmission** |  |
|  |  | Describe the storage area of PI |
|  |  | If involving the cross-border transfer of information, state the standards, agreements, and legal mechanisms to be followed |
| **Information destruction** |  |  |
|  | **Storage time limit** |  |
|  |  | State the retention period of PI |
|  |  | Delete or anonymize PI after the deadline |
|  | **Data deletion and anonymization** |  |
|  |  | Delete or anonymize PI after account cancellation |
|  |  | Notify third parties to promptly delete PI |
| **Rights of PI subjects** |  |  |
|  | **Inquiry of PI** |  |
|  |  | Right to inquire PI |
|  |  | Provide methods to inquire PI |
|  | **Correction of PI** |  |
|  |  | Right to correct PI |
|  |  | Provide methods to correct PI |
|  | **Deletion of PI** |  |
|  |  | Circumstances that PI subject has right to delete PI |
|  |  | Provide methods to delete PI |
|  | **Withdrawal of consent** |  |
|  |  | Right to withdraw consent |
|  |  | Provide methods to withdraw authorization |
|  |  | No longer process the corresponding PI after the withdrawal of consent |
|  |  | Right to refuse to receive business advertisements delivered based on PI |
|  | **Account cancellation** |  |
|  |  | Right to cancel account |
|  |  | Provide methods to cancel account |
|  |  | If manual processing is required, complete verification and account cancellation within 15 working days |
|  | **Obtain copies of PI** |  |
|  |  | Right to obtain copies of PI |
|  |  | Provide methods to obtain copies of PI |
|  | **Responding to requests** |  |
|  |  | Respond to PI subject requests within 30 days or the time limit of the laws |
|  |  | Circumstances that allow no need to respond to PI subject requests |
|  | **Complaint management** |  |
|  |  | Limits on information system automated decision making |
|  |  | Provide means to lodge a complaint |
|  |  | Answer within the time limit prescribed by laws and regulations |
|  |  | Provide means to resolve the issue through external parties. |
